# Slow Burns: A Qualitative Study of Burn Pit and Toxic Exposures Among Military Veterans Serving in Afghanistan, Iraq and Throughout the Middle East

**Published:** 2021-12-27

**Authors:** Pollie Bith-Melander, Jack Ratliff, Chelsey Poisson, Charulata Jindal, Yuk Ming Choi, Jimmy T Efird

**Affiliations:** 1 Department of Social Work, California State University, Stanislaus, Turlock, CA, USA; 2 Department of Medical-Surgical Oncology, James A Haley Veterans Affairs Hospital, Tampa, FL, USA; 3 Military Exposures Team, HunterSeven Foundation, Providence, RI, USA; 4 Harvard Medical School, Harvard University, Boston, USA; 5 Signify Health, Dallas, TX, 75244, USA; 6 Cooperative Studies Program Epidemiology Center, Health Services Research and Development, DVAHCS, Durham, USA

**Keywords:** Burn pits, Oil well fires, Military exposures, Explanatory models, Qualitative analysis, Deployment anthropology

## Abstract

During deployment to the Persian Gulf War and Southwest Asia theatre of operations, Veterans often experienced various hazards, foremost being open-air burn pits and oil well fires. While over 23 presumptive conditions (ranging from brain cancer, interstitial lung disease, and lymphomas to sleep/mood disorders, depression, and cognitive impairment) have been studied in connection with their military-related exposures, there is a paucity of qualitative research on this topic. This is especially true in the context of explanatory models and health belief systems, vis-à-vis underlying social and cultural factors. The current paper provides a balanced conceptual framework (summarizing causal virtues and shortcomings) about the challenges that Veterans encounter when seeking medical care, screening assessments and subsequent treatments.

## Introduction

The preliminary results from our study about the exposure to burn pits and environmental toxins from the war in Iraq published in April 2020 entitled “A Pilot Study of Airborne Hazards and Other Toxic Exposures in Iraq War Veterans” suggests that further exploration into this topic is critical in gaining a deeper understanding of the origins and causes for negative health outcomes among Veterans of recent wars [1]. Some of these Veterans, as well as local civilian populations, were exposed to both environmental and man-made chemicals and toxins, especially the more hazardous airborne variety. Airborne toxins represent the main class of exposures reported by men and women who served in conflicts in locations throughout the Middle East including both Iraq, and Afghanistan. This includes particulate matter from aviation and diesel exhaust fumes, combat-related smoke from ground ordnance and air strikes, dust storms, on-base contact with open-air burn pits, and oil-well fires [2].

Our publication was the first health outcomes survey of Operation Iraqi Freedom (OIF) Veterans conducted by a Veteran peer-support organization. A critical and important finding from this research was that those Veterans who served in support of OIF were potentially exposed to various airborne toxins that consequently manifested increased respiratory-related symptoms leading to a decrease in overall physical fitness status [1]. We also examined the Veterans’knowledge of exposure to burn pits and other toxins while deployed to explain the negative health outcomes faced post-deployment.

A significant decline in overall health following deployment raises concern as to the role of toxic exposures among Veterans who have served throughout the Middle East in support of these wars, especially an increase in early onset malignancies, rare diseases, and respiratory-related symptoms across all gender lines. A deeper understanding of the role of exposure to burn pits and other combat-related toxins, including their proximity to and involvement with the exposure, can offer valuable insight into the ongoing medical challenges, diagnostics, and treatments for this growing, unique Veteran population.

Like military Veterans in other conflicts, including those of Operation Desert Shield (ODSh), Operation Desert Storm (ODSt), Operation Iraqi Freedom (OIF), and Operation Enduring Freedom (OEF) Veterans were exposed to a variety of hazardous and potentially toxic agents and psychological stressors, leading to increasing reports of complex medical problems and ill-defined symptoms. Some of these adverse effects are acute in nature while others become chronic with long-lasting impacts. The most noticeable were respiratory health conditions including asthma, bronchitis, chronic obstructive pulmonary disease, sinusitis, and lung cancer, as well as rare and unexplained illnesses and cancers [3,4].

A “burn pit” refers to a constructed hole in the ground routinely used to dispose of environmental garbage and military waste. Burn pits have been used as a common practice by the United States military for more than two decades as a means of eliminating solid waste in a timely manner while maintaining operational security. Reports over the last twenty years of conflict in the Middle East state there were approximately 153 burn pits in Iraq and 99 in Afghanistan, while mobilization-stations in Kuwait and Uzbekistan had over 25 functioning burn pits [5]. Considering ongoing engagements in the special operations communities, bases located in Africa, Jordan, and Syria, burn barrels, and burn pits, are commonly used as a rapid means of waste disposal. Additionally, reports have surfaced that surrounding United States and partner force military instillations around the world (Africa, Djibouti, Doha, Egypt, Haiti, Jordan, Kyrgyzstan, Kosovo, Pakistan, Qatar, Saudi Arabia, Somalia) also utilize this method of waste disposal. More than 4.2 million military personnel have been deployed to Afghanistan and Iraq [6] and potentially exposed to the smoke and soot from burn pits. Burn pits were originally developed as a temporary solution for managing solid waste, but they have evolved into routine practice owing to their low costs and ease of operation. The main reason for burning instead of storage involves environmental considerations [7]. The disposal of waste is said to help minimize the risk for contamination of environmental media, including ground water. Perhaps the need to remove waste from military bases efficiently is another motivation for burn pits. The amount that has been burned is staggering. For example, the amount of solid waste burned at Balad Air Base was estimated at approximately two-hundred tons of solid waste per day during peak years (2005, 2007) of Operation Iraqi Freedom [7] which occupied roughly 10 acres [8]. The Balad Air Base burn pit burned chemicals, incomplete combustion by-products, medical and human waste, metal, munitions, plastics, petroleum and lubricant, rubber, Styrofoam, other unexploded ordnance, and treated wood [8] and was often ignited using the benzene-based Jet fuel Propellant (JP-8) as an accelerant [9]. This agent adheres to contact surfaces for a longer period than standard petroleum-based fuels, extending the health risk of exposure [10,11] from both an airborne inhalation and absorption-based exposure.

An air sampling analysis was performed for the Balad base in Iraq toward the end of military operations [12]. The air sampling detected Particulate Matter (PM), Polycyclic Aromatic Hydrocarbons (PAHs), Volatile Organic Compounds (VOCs), and toxic organic halogenated dioxins and furans (dioxins). Particulate Matter (PM) is a complex mixture of extremely small particles and liquid droplets. PAHs are a group of over 100 different chemicals that are formed during the incomplete burning of coal, oil and gas, garbage, or other organic substances like tobacco and charbroiled meat [13]. They are usually found as a mixture containing two or more of these compounds, such as soot [13]. They usually exist as colorless, white, or pale yellow-green solids. PAHs are found in coal tar, crude oil, creosote, and roofing tar, but a few are used in medicines or to make dyes, plastics, and pesticides [13]. VOCs are emitted as gases from certain solids or liquids. These include chemicals such as acetone, benzene, chlorodifluoromethane, ethylbenzene, dioxins etc., and are known to be associated with herbicide use in Vietnam [13]. In other words, what was burned was anything that was considered waste; whether it is biological material, gasoline, oil, plastics, and tires etc. Inhalation of the fumes produced by burning such waste appears to contribute to the development of various medical disorders.

## Explanatory Models of Illness

This qualitative analysis is framed using explanatory models of health and illness. To date, most studies have been topical in nature, focusing on the physiological consequences of exposures to burn pits and toxins. Accordingly, there is yet a qualitative research study to address patients’ explanatory models of illness. The theory of Explanatory Models (EMs) proposes that individuals and groups can have vastly different notions of health and disease [13]. EMs is defined as patients’ understanding and beliefs of illness and seeking treatments based on these beliefs. The medical providers’ explanatory models of illness are generally based on the biomedical model, which emphasizes the biological and physiological aspects of disease etiology [14]. However, patients may not follow up on the medical provider’s course of treatment and/or recommendations. Instead, patients and individuals who are experiencing illness may have different explanatory models, and this difference in belief systems can hinder the course of treatments and disrupt health outcomes. Studies that have explored variations in these models have found that explanatory models of illness are influenced by social and cultural contexts and prior experiences and perceived notions [15]. Three major concepts are frequently interrelated within practice to determine the connection between the person, environment, and health, defined as the degree of wellness or well-being that the Veteran experiences, the clinical capstone of Florence Nightingale, and the importance of a community/cohorts understanding [16].

The explanatory models and their etiologies are embedded in people’s beliefs. They reflect cultural theories of illness and treatment. The experiences, exposure to various systems, cultures, values, and education of specialists also influence the type of medical services they provide. A strong healing motivation can contribute to a patient’s psychological and physical well-being, and thus lead to improvement in the patient’s health [17]. Treatment usually follows the advice of professionals, family members, or friends. These individuals play an important role in how patients manage illness or maintain health. Treatment decisions are influenced by explanatory models of illness and health and beliefs about different types of therapies. Gaining a deeper understanding of beliefs and experiences about illness and symptoms may offer insight into treatments and interventions, as well as increased effectiveness in therapeutic efficacy.

Treatment decisions can be strongly influenced by professionals, family, or friends. A deeper understanding of the patients’ beliefs and experiences about illness and symptoms may offer insight into strategies to improve patient compliance with prescribed therapies.

Explanatory models of diseases and their etiologies are embedded in people’s beliefs and therefore the actions that follow. Anthropologists have long understood that any effort to change human behavior rests on studies that address questions of why people behave as they do. Such studies should place an emphasis on the social, cultural, and psychological aspects of human health and illness, particularly beliefs about etiology, diagnosis, and efficacy. Programs intended to combat disease can be met with resistance if individuals’ explanatory models of illness are ignored. Ethnomedical beliefs about diseases are not always congruent with the biomedical paradigm, as some scholars have shown [18]. Two aspects of health belief systems can be analyzed. First, societies actively change the local ecology to increase or decrease the risk of certain diseases [19]. Second, culture provides a theoretical system for understanding and attempting to manipulate through medicine the diseases that cause human suffering and death [19]. By understanding both aspects, medical anthropologists can provide data that improves the effectiveness of intervention programs, particularly for symptoms that have yet to be labeled and understood by medical professionals and their role in the prevention interventions. What is needed is an in-depth analysis of Veterans’ beliefs about treatment efficacy, illness etiology, prognosis, and Nosology of exposure to burn pits and other combat-related toxins within their own ethnomedical systems.

One of the main challenges in understanding a new phenomenon such as Gulf War Syndrome (GWS) for example is that it lacks the fundamental clarity on the classification of the illness or the disease. Disease is biologically defined, which has a biomedical construct as a base for understanding an illness experience. Illness is a cultural construct that considers subjective and personal experiences in alignment with objective symptomology. All healthcare providers are still on a steep learning curve to understand this new phenomenon and to provide individualized, holistic healthcare. The combined lack of appropriate or effective care and the ambiguities in patients’ explanatory models of illness further create confusion for both those Veterans who seek medical advice and those who provide medical care.

The two additional conceptual frameworks that must be defined to fully grasp the nature and extent of the problem of this illness category are etic and emic. An ambiguous illness category such as Gulf War Syndrome and its related health conditions can best be understood by these terms. The biomedical models focused on in clinical settings measure disease processes as the outcome, assuming an etic view, while some alternative models such as those used by medical anthropologists emphasize quality of life and life duration instead of disease process with an emic perspective [20-22]. Alternative models have been developed in response to the observation that not all patients adhere to the recommendations offered by medical providers, even if they were part of the decision-making process [22]. An emic view considers cultural and social factors. Cultural, religious, and emotional factors contribute to decision-making process, as do life experiences and health status [23]. Such models emphasize identifying non-medical factors as they influence decisions about treatment [24]. Non-medical factors are divided into three categories: Characteristics of the patient (e.g., age, sex, socio-economic status, race or ethnicity, presence and type of health insurance, personality characteristics, and physical attractiveness); characteristics of the doctor (e.g., medical specialty, level of training, length of clinical experience, geographical location, and age, sex, race, ethnicity, and personality); and features of the practice setting (e.g., organization of the practice and cooperativeness of the physician) [24]. All these characteristics influence the way patients make medical decisions. Health decisions are also made based on cultural themes such as language, beliefs, and kinship systems [25]. Individuals also make decisions about treatment based on personal explanatory models guided by previous responses to illness episodes [26]. Recognition of the importance of the role of the individual in making treatment decisions is therefore essential. What people believe and the actions they take in dealing with illness are an important part of the process of how medical decisions are made.

## Role of the Individual in Medical Decision-Making Process

Medical anthropologists have broken away from testing specific medical models in the clinical setting to debate normative and descriptive approaches to understanding decision-making. Both the normative and descriptive treat individuals as rational decision-makers but diverge with respect to the role of the individual in the decision-making process.

The normative approach predicts human behavior by assuming that people rationally evaluate all alternatives and makes the best choices following a mathematical model [27]. This implies that the process by which individuals’ reason should and does emulate mathematical calculations. The optimal decision generated by the normative model is supposed to reflect the decisions that people make in the real world [27].

The descriptive model considers what individuals say and do. This approach arose as a reaction against the normative approach [27]. Anthropologists following this model pay greater attention to the day-to-day actions of people confronted with illness and attempts to gain insight into the relationship between cultural knowledge and specific treatment actions [20,21]. The descriptive theory assumes that people in the real world do not often make the optimal choices predicted by normative modeling [27]. Instead, it attempts to account for actual choices people make in their natural settings. Understanding the cognitive processes that underlie choice-making improves the likelihood of predicting people's actions accurately [27]. Garro [20,21] defines the decision-making process as an inclusive “higher-level category” used to evaluate the subcategories of actual options. Garro [20,21] further treats rationality as everyday logical and self-benefiting behavior where people make daily decisions, rather than as a mathematical process.

Decisions about health care are affected by characteristics of household members, including age, gender, and sex. In general, adults in the family play a central role in making more of the decisions concerning their children's health and accessing health care services. If messages from the medical professionals are incongruent with the individual’s experience. Patients may signal their approval and simultaneously withhold their doubt or disagreement. Other factors such as socio-cultural themes, including educational level and background, can be driving this behavior, including beliefs about disease causation, the pragmatics of the situation, Familism, language (if English is native), and other factors.

Currently, what constitutes Gulf War Syndrome points out that the emic classification is not necessarily in line with its own biomedical definition, which is based purely on etiology? Each emic class may not be a distinct etic illness. For example, different stages of an illness sometimes fall into different emic categories as the symptoms change. Sometimes one category encompasses multiple illnesses if they all share the same causative agents. Despite several well-funded government research studies, GWS is still lacking in definition and poorly understood.

## Gulf War Syndrome

Gulf War Syndrome (GWS) is a cluster of medically unexplained chronic symptoms, including dizziness, fatigue, headaches, indigestion, insomnia, joint pain, memory problems and respiratory disorders. It is a widely used term to refer to the unexplained illnesses occurring in Veterans of the 1991 Gulf War. The Department of Veterans Affairs refers to these illnesses as "chronic multisymptomatic illness" and "undiagnosed illnesses" (United States Department of Veterans Affairs, 2013). Some of the possible causes include chemical warfare agents (e.g., nerve gas, or pyridostigmine bromide, which was given as a preventive measure to soldiers likely to be exposed to chemical warfare agents) [28]. Another potential cause is psychological such as Post-Traumatic Stress (PTS) and anxiety spectrum since Veterans with Gulf War syndrome symptoms have high rates of accompanying psychiatric disorders [28]. Additional factors to consider are exposures to toxic waste and environmental factors. Some Veterans might have been exposed to other chemical agents, such as corrosive liquids, depleted uranium, pesticides, and smoke from oil well fires, solvents, and heavy metals that were used during repair and maintenance.

## Direct Link of Exposure to Burn Pits: Bronchiolitis

According to the Morbidity and Mortality Weekly Report [29], Respiratory Syncytial Virus (RSV) is the most common cause of Lower Respiratory Tract Infection (LRTI), especially found in young children worldwide. Approximately half of all LRTI-associated hospitalizations are caused by bronchiolitis [30]. Bronchiolitis is an infection of the bronchial and bronchiolar epithelial cells, with subsequent inflammation and edema that results in airway obstruction. This process manifests into clinical symptoms of coughing, respiratory distress, wheezing and tachypnea. Constrictive bronchiolitis, however, is a primary disorder of the bronchioles in which inflammation, smooth muscle hypertrophy, and/or fibrosis leads to narrowing of the lumen [12]. Lumen is the cavity or channel within a tube or tubular organ such as a blood vessel or the intestine. It is associated with chronic lung transplant rejection, graft *vs.* host disease in allogeneic hematopoietic cell transplantation, collagen-vascular and inflammatory bowel disease, drugs, gastroesophageal reflux, healed infections, microcarcinoid tumor lets, and inhalation of mineral dust or toxic fumes [31]. Constrictive bronchiolitis in otherwise healthy individuals is rare and often difficult to diagnose [31]. The condition is well-documented in a study conducted by King et al. who reported the diagnosis of constrictive bronchiolitis in 38 previously healthy soldiers recently returned from service in Iraq and/or Afghanistan [12]. All the patients required a surgical lung biopsy to establish the diagnosis after extensive non diagnostic noninvasive evaluation [12].

Since the start of conflicts in Afghanistan and Iraq, approximately 2.4 million troops have been deployed in Operation Enduring Freedom (OEF) and Operation Iraqi Freedom (OIF) [32]. Most were likely to be exposed to deployment locations that include large arid or semiarid regions where there is frequent exposure to desert dust and sand [33]. In addition, those deployed to Afghanistan and Iraq might have experienced high levels of find Particulate Matter (PM) and the varied exposure attributable to military operations such as burn pit emissions from open-air waste burning, vehicular exhaust, and other poor-regulated industrial point sources [33]. Perhaps owing to this high level of exposure to these environmental variables, including military practices of removing waste through burning in open air, created a perfect storm to shift a healthy body to a sick one in a short period of time (i.e., one deployment). In a sample of 771,874 who utilized VA healthcare, about 200,000 Veterans reported diseases related to the respiratory system [32], and the majority were diagnosed with respiratory symptoms (including cough and dyspnea). Another study surveyed military personnel immediately after leaving deployment to Iraq or Afghanistan from 2003 to 2004, and of 1,250 self-reported, 19% reported wheezing without a history of asthma after deployment compared with 6% of those pre-deployment [34]. This followed with respiratory illnesses at 69% [34]. There is a historical trend of troops who reported respiratory and asthma symptoms immediately after deployment. For example, surveys of Soviet troops from the 1979-1989 war in Afghanistan found that 43% of service personnel had bronchitis and/or pneumonia within the first year in Afghanistan [33].

## Research Methodology

This paper utilized secondary data from a sample of ~2,000 participants collected with two questions focused on qualitative responses [1]. Both questions prompted participants to make comments as to what was happening to them/others and what they had to say about their experiences. Through these questions, we were reviewing for specific data relating to the following key questions:

- What do military service members/Veterans think happened to their bodies? In other words, what are the explanatory models of illness from exposure to burn pits?
- What are some of the symptoms they have experienced since being exposed to burn pits?
- What challenges do they face when they seek treatments?
- What treatments have they received since returning from deployment?

## Results

Results indicated some general patterns of challenges, including lack of sleep and basic needs (e.g., clean water, protected shelter, etc.), long daily work hours, immediate changes in body and health, and development of apparent symptoms. A major theme is that all parts of a human body are impacted.

Specifically, these various parts of the body include the brain, throat, organs, skin, bone, and nose. The impacts took place during deployment and post. Some stated having cysts, lymphatic issues, and cancers as the diagnoses made by either Veterans Affairs medical providers or civilian providers following their deployments. Other comments of sickness or health issues are tabled in Table 1.

## Data Analysis

The impact of toxic exposure during military service appears to contribute to a broad range of illnesses. Respondent’s perceptions of symptom etiology are related to burn pit and toxic exposures. Almost all body parts including blood, different organs, and tissues are affected, and developed symptoms as perceived by the participants from the exposure to burn pits where they were stationed. The general patterns the participants stated include chronic breathing difficulties, fatigue, general pain, memory issues, slow recovery, and sleep issues. Additionally, sensitivity to light and problematic skin conditions such as rash and chronic infections.

Another significant finding is the participants’ knowledge of how they have become sick. Participants seemed to recognize that there is a direct link between exposure during deployment and their health outcomes. Some stated that they felt some of the symptoms as early as one month into the deployment.

Various parts of the body and brain were impacted and manifested in physiological symptoms. Brain, blood, heart, lung, skin, and vital signs are some of these examples. The more subtle changes are those such as rare cancers, lymphatic swelling, and sensitivity to light. One significant comment seen throughout the responses is slow recovery during post deployment. In addition, some concluded that something happened with their immune systems after deployment. Examples cited by these participants included general pain, fatigue, and sensitivity. They noticed health was different post deployment. In other words, their bodies changed for the worse as the result of fatigue and slow recovery when they experienced a cold or flu.

Those who responded with multiple deployments claimed that they ignored their bodies’ responses to the first deployment out of fear and hoped that whatever impacted or changed would “just go away at some point or heal itself”. Time and distance from services also play a role in how they responded during deployment. One example was that one participant said that they knew that something was not right in their body after the first deployment; however, the services were too far from where the person was living at the time. In addition, the participant said that there was the issue of preparing for another deployment. The person was worried that any health issue would lead to disqualification.

Another example was seeing too many experts who offered contrasting opinions, which caused further confusion. The person said that both Veteran doctors and those who worked outside of the VA system seemed not to know what to do with their health condition. One comment was about disillusionment about the U.S. healthcare system. The person felt betrayed by the system, but the person was worried about the lack of support for the family when the person is no longer around because of death from ill health. Another respondent stated that the exposure during deployment was linked to ill health.

Overall, what appears to be common is slow recovery and difficulties in breathing after one deployment to these sites as noted in 83% of Operation Iraqi Freedom Veteran respondents [1]. In addition, the participants seemed to recognize the change in health before and after deployment. Most noticeable is the rapid decline of health with a lack of history of health problems, which begs the question what happened during the deployment period to cause such a rapid deterioration of health after less than a year. There are multiple factors that might have contributed to rapid declines in health, including both environmental and burn pit exposure. Furthermore, jetlag, food, or water that the body is not used to, as well as rigors in work schedule with long hours without adequate rest periods can exacerbate health conditions if the body is already compromised by environmental exposure such as sandstorms, heavy metals in the soil, and fumes or other particles from burning anything and everything on military bases. This is in conjunction with the lack of sleep and increased stress level in theater, which would certainly guarantee the likelihood of slow recovery from a cold or flu or other infections. Being away from loved ones and feeling strange being in a foreign land can hinder one’s ability to cope in an already stressful situation. In other words, the body might not have time or a chance to heal from even some of the minor but common health challenges such as a cold.

## Limitation

One limitation of this manuscript is the lack of data from observations or direct interviews. In addition, no probe was conducted as a follow-up. It is limited to voluntary responses, which consisted of participants being asked if they had anything else to say regarding their experiences. Some suggested methods for future research studies include other types of qualitative data such as observations and open-ended interviews with an interview guide to gain a deeper understanding of the locations, whether there was direct/indirect exposure to burn pits, and wind directions. It would also be important to ask about environmental and other related factors for exposure. In addition, some follow-up on questions of basic needs would be critical beside experiences such as lack of sleep and long work hours without breaks. The importance of military occupational specialties (specifically special operations) and operational tempo have on exposure trends and subsequent health conditions would be beneficial to consider for future research.

## Conclusion

Manifest were the participants’ knowledge of the link between war exposure and health outcomes, particularly those related to both environmental factors like sandstorms and man-induced factors of military-associated practices of getting rid of waste on military bases through burning. What happened to change a young healthy body to a rapidly aging one after a single deployment perhaps could be explained by knowing the experiences of soldiers in war zones?

Veterans whose symptoms appeared during post-deployment recognized immediately the link of their exposure to their health outcomes or illness that they experienced from deployment to locations in Afghanistan and Iraq. While they might not have known what diseases or illnesses they were experiencing, they were able to recognize the deterioration of their health owing to slow recovery or apparent symptoms, which exacerbated rapidly once diagnosed by a medical professional. They also noticed the way they felt was different during post-deployment and recognized that something happened to them while they were in theater.

The environmental, man-made, and psychological factors (including stress) are worth exploring further to determine the direct link of sickness and health with these military personnel. Furthermore, it would be useful to explore what is required in pre-deployment preparedness practices that might impact a human body such as vaccination and other health prevention medications that are prescribed during the deployment, including pain medications, antibiotics, sleeping pills or other medications for managing emotions (anxiety spectrum, depression, or any other psychiatric issues).

## Figures and Tables

**Table 1:** Comments of sickness or health issues.

**Neurological**
Migraines
Insomnia
Vertigo
Sensory disruptions (i.e., blurred vision, tinnitus, taste/smell)
**Cognition**
Poor concentration
Mood changes, depression
Recall disruption
Word and sentence formation
**Respiratory-related**
Cough (productive and dry)
Chronic sinusitis, rhinitis, bronchitis
Lung-tissue scarring
Asthma
Shortness of breath
**Immune System**
Autoimmune conditions (unknown)
Chronic state of inflammation
**Cancers**
Skin (Melanoma, Basal Cell)
Reproductive (Cervical, testicular, uterine, ovarian)
Lymphoma (Non-Hodgkin’s, Hodgkin’s, Mantle cell)
Leukemia (Myeloid, Myeloma, Lymphoblastic)
Breast (ductal, in situ DCIS)
Renal (Bladder, kidney)
Endocrine (Thyroid, Pancreatic)
Brain (Glioblastoma)
Gastrointestinal (colon, rectal, stomach, bile duct, esophageal)
Connective tissue & bone (Sarcoma, Rhabdomyosarcoma, Ewings)
Lung (small-cell, sarcoma, mesothelioma, bronchus, intrathoracic)
**Skin**
Vitiligo
Blistering
Chronic infections/prolonged healing
Psoriasis
Dermatopathy lymphadenitis
Dermatopathy lymphadenopathy
**Exposure**
Heavy metals (Arsenic, Chromium, Copper, Lead, Mercury, Sulfur, Uranium)
Human waste/bodily fluids
White phosphorus (mortar/ordnance), mustard gas, Sarin, Depleted Uranium
Smoke and Soot from burning waste, explosions, IED/VBIED/RPG blasts, leaded gasoline, local pollution, small particulates from dust storms
Fumes from heavy machinery, aviation, military vehicles
Asbestos from local housing
Sulfur and lead from gunfire
**Qualitative, Subjective Experiences (During, Post-Deployment)**
Got sicker and slower to recover
Nose/throat felt not right, and I stayed very close to the burn pit
Having difficulty breathing
Everything was bad. Food. Smoke. Dust. Sandstorms almost all the time.
All of us felt sick. From smoke. From food. Not sleeping. Not eating. Vomiting and nauseous.
Live too far to get help
Didn’t know what was happening to me when I returned home after my first deployment
Feeling sicker at home
Feeling sick after about one month on base in Ballad during first deployment
The smell was so bad that my nose felt numb
Worried about my children when I am gone
Bases caused ill health/diseases
Chemical exposure was the key behind the illnesses
Too confused about experts’ opinions and uncertain how to make decisions
Cannot pinpoint the problem, but the body is not right
I am feeling weak
I cannot run anymore after my first deployment
I am having hard time breathing, and this was not the case before my first deployment.
I tried to ignore it even though I knew something was not right with me
My doctor gave me so many meds, for my pain, for insomnia, for PTSD, for headaches, for nausea
I didn’t know where to go and get help even when I was not healing properly. My cold lasted months
I worked long hours every day, 16-plus per day
I slept in a sleeping bag the first few weeks when I got to Balad
It was hot, and lots of sandstorms
I inhaled dust and sand during the first deployment
